# Nervous system examination on YouTube

**DOI:** 10.1186/1472-6920-12-126

**Published:** 2012-12-22

**Authors:** Samy A Azer, Sarah M AlEshaiwi, Hala A AlGrain, Rana A AlKhelaif

**Affiliations:** 1 Professor Medical Education and Chair of Curriculum Development & Research Unit, College of Medicine, King Saud University, P O Box 2925, Riyadh, 11461, Saudi Arabia; 2 Department of Medical Education, College of Medicine, College of Medicine, King Saud University, P O Box 2925, Riyadh, 11461, Saudi Arabia

**Keywords:** YouTube, Medical education, Nervous system examination, Web-2, Learning resources

## Abstract

**Background:**

Web 2.0 sites such as YouTube have become a useful resource for knowledge and are used by medical students as a learning resource. This study aimed at assessing videos covering the nervous system examination on YouTube.

**Methods:**

A research of YouTube was conducted from 2 November to 2 December 2011 using the following key words “nervous system examination”, “nervous system clinical examination”, “cranial nerves examination”, “CNS examination”, “examination of cerebellum”, “balance and coordination examination”. Only relevant videos in the English language were identified and related URL recorded. For each video, the following information was collected: title, author/s, duration, number of viewers, number of posted comments, and total number of days on YouTube. Using criteria comprising content, technical authority and pedagogy parameters, videos were rated independently by three assessors and grouped into educationally useful and non-educationally useful.

**Results:**

A total of 2240 videos were screened; 129 were found to have relevant information to nervous system examination. Analysis revealed that 61 (47%) of the videos provided useful information on the nervous system examination. These videos scored (mean ± SD, 14.9 ± 0.2) and mainly covered examination of the whole nervous system (8 videos, 13%), cranial nerves (42 videos, 69%), upper limbs (6 videos, 10%), lower limbs (3 videos, 5%), balance and co-ordination (2 videos, 3%). The other 68 (53%) videos were not useful educationally; scoring (mean ± SD, 11.1 ± 3.0). The total viewers of all videos was 2,189,434. Useful videos were viewed by 1,050,445 viewers (48% of total viewers). The total viewership per day for useful videos was 1,794.5 and for non-useful videos 1,132.0. The differences between the three assessors were insignificant (less than 0.5 for the mean and 0.3 for the SD).

**Conclusions:**

Currently, YouTube provides an adequate resource for learning nervous system examination, which can be used by medical students. However, there were deficiencies in videos covering examination of the cerebellum and balance system. Useful videos can be used as learning resources to medical students.

## Background

Introducing problem-based learning (PBL) into most medical curricula worldwide and emphasising student-centered learning and self-directed learning has caused significant changes in the teaching and learning pedagogy. Students enrolled in such courses use a wide range of resources as they prepare for their self-directed learning and learning issues. These learning resources include review papers, journal articles, textbooks, museum specimens, simulated patients, computer-aided learning programs and multimedia [1]. However, recently it has been shown that medical students use Google and YouTube as the first resources for their research [2]. YouTube has shown some promise as a learning resource to students and the general public.

YouTube was created in 2005 as an arena for personal communication, social networking and for distribution of commercial content. Within the first few days of the 2011 Egyptian uprising, Internet and YouTube was used as a mean of social communication for safety and progress of the uprising [3]. Although similar services such as MetaCafe, Yahoo Video, and Google Video, Vimeo, DropShots, and others are available for public use, YouTube has become the most popular video-sharing service worldwide [4,5]. Statistical data offered by YouTube provide evidence of its popularity. For example, YouTube is the third most visited website on the Internet after Google and Facebook. Also, YouTube is the first largest video site with over 100 million visitors per month, and more than 65,000 new videos being uploaded everyday making approximately 360 hours of videos uploaded every 24 hours [4-6]. The reasons for the popularity of YouTube can be attributed to a number of factors including: the relative ease of uploading and sharing videos, and the continuous improvement in the design of the YouTube site to reflect the evolution of online social networking and enhance its use for commercial content, broadcasting networks, movie studies, political changes, and education. In fact, YouTube has succeeded in providing social networking and enabling discussion among viewers. YouTube has also encouraged the sharing and embedding of videos in other social media such as blogs, wikis, and emails.

Videos have the advantage of explaining difficult concepts through using simulation, graphic diagrams, dynamic illustrations, analogies, and simulated patients. The teaching/learning benefits of videos will be enhanced if videos are well designed, explore scientifically correct content, clearly presented, and address learners’ need. Researching for educationally useful videos on YouTube may be time consuming and requires knowledge from researchers about what makes an educationally useful video. Recently, YouTube videos have been evaluated in a number of areas related to medical/health issues such as information on immunization [7], human papilloma-virus vaccination [4], prostate cancer [8], H1N1 influenza pandemics [9], rheumatoid arthritis [10], as a learning resource in nursing [11-13], surface anatomy [14], cardiopulmonary resuscitation [15], dental education [16] and as a patient resource for infantile spasms [17].

However, there is no study assessing the usefulness of YouTube covering examination of the nervous system and whether the video clips on YouTube are authentic and educationally useful for learning such an examination. This study aims at assessing YouTube videos covering nervous system examination.

## Methods

During the period from 2 November to 2 December 2011, YouTube (http://www.youtube.com) was researched for videos covering nervous system examination. The following key words, “nervous system examination”, “nervous system clinical examination”, “cranial nerves examination”, “CNS examination”, “examination of cerebellum”, “balance and coordination examination” were used in the research. Only relevant video clips in the English language were identified and the related URL was recorded. For each video the following information was collected: title, duration of the video, number of days on YouTube, total number of viewers, name of uploader/creator (organization, group of people, one person), and number of comments made by viewers (Table 1). Videos were then evaluated using criteria targeting content, technical, authority, and pedagogy parameters. As per our previous study with some modifications [14], items covering these parameters were grouped under major and minor criteria. Major criteria comprise: (i) Contents about clinical examination are scientifically correct, (ii) Images are clear, (iii) Creator and/or organization providing the video are mentioned, (iv) Topic is clearly presented, and (v) Sounds are clear and background is free from noise. The minor criteria comprise (i) Video covers topic identified in the title, (ii) Designed at the level of undergraduate medical/health science students, (iii) Time to download is reasonable, (iv) Information about the creator is up-to-date, and (v) Educational objectives are stated, and (vi) The video uses simulated patients or patients to demonstrate the examination (Table 1).

**Table 1:** Data collected for each video clip and assessment criteria

Data collected:	
	- Title, duration of the video, number of days on YouTube,
	- Total number of viewership.
	- Name of creator (organization/group of people/person)
	- Any links to the video.
	- Number of comments of viewers and comments made
Assessment criteria:	
Major criteria:	
	1. Contents about clinical examination are scientifically correct
	2. Images are clear.
	3. Creator and /or organization producing the video are mentioned.
	4. Topic is clearly presented.
	5. Sounds are clear and background is free from noise.
Minor criteria:	
	1. Covers topic identified.
	2. Designed at the level of undergraduate medical/health sciences students.
	3. Time to download is reasonable.
	4. Information about the creator is up-to-date.
	5. Educational objectives are stated.
	6. The video uses simulated patients or patients to demonstrate the examination.

Educationally useful videos should fulfill all major criteria and at least three other items under the minor criteria. Two scores were allocated for each item in the major criteria, and one score was allocated to each item under the minor criteria. If an item was fulfilled, allocated score was given; if not, a zero was given. No half scores were used. If videos were placed on YouTube under 2 or more parts, each part was examined on its own and assessors treated them as one video as they apply the criteria. A useful video should score 13 or higher.

Two scores were allocated for each item in the major criteria and one score was allocated to each item under the minor criteria. If an item was fulfilled an allocated score was given; if not fulfilled, a zero was given. No half scores were used. Educationally useful videos shall fulfill all major criteria and at least three items under the minor criteria. To standardize the evaluation of contents and the process of nervous system examination, the assessors used the video provided by Talley and Connor’s textbook [18] as a reference for their assessment.

Prior to applying the criteria we piloted its use. It is worth to note that in our initial trials of testing the criteria, we placed the item “uses simulated patients or patients to demonstrate the examination” under major criteria. However, this item was of low discrimination as it was fulfilled in all videos finalised (129 videos), see Table 1. Therefore, we moved this item to the minor criteria. The same applies to the item “Time to download is reasonable”. It was of low discrimination and kept under minor criteria. The criteria were applied by three assessors (SMA, HAA, and RAA). None of the assessors shared their findings or discussed the outcome of their evaluation. An Excel sheet covering the three evaluations was examined by a fourth researcher (SAA). The agreements among the assessors were in the range of 96-98%. The findings were discussed among the researchers. The criteria items were tested again by three assessors for another 25 videos. The evaluation showed that the agreements between assessors were in the range of 98-99% indicating the reproducibility of the results among the assessors.

Videos were then rated independently by three assessors (SMA, HAA, and RAA). Videos that were difficult to classify or, when there was a disagreement among assessors, all researchers reviewed such videos in a meeting and reached a final agreement. Data were entered on Excel sheets and were checked before conducting any analysis. All analysis was performed using SPSS for Windows 14.0 (SPSS Inc., Chicago, IL, USA), version 16 software.

## Results

**Figure 1:**
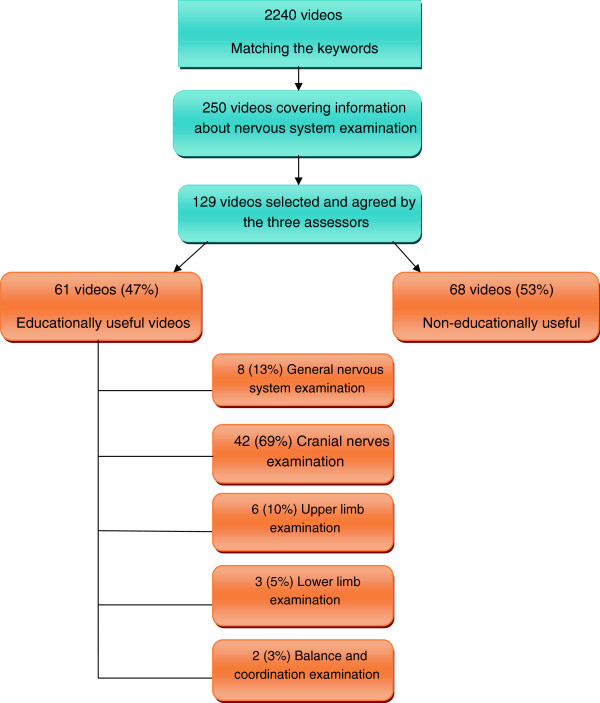
Screening YouTube for eligible videos.

A total of 2240 YouTube videos were screened and 129 were found to have relevant information to clinical examination of the nervous system. The total duration of these video clips was 490 minutes (Table 2). The use of the criteria for grouping the videos to useful videos and non-useful videos revealed that there were 61 videos (47%) that provided useful information on clinical examination of the nervous system (Table 2), and scored (mean ± SD, 14.9 ± 0.2). 8 videos (13%) covered general examination of the nervous system, 42 videos (69%) covered cranial nerves examination, 6 videos (10%) covered upper limb examination, 3 videos (5%) covered lower limb examination and, 2 videos (3%) covered balance/coordination examination. The total duration of these videos was 253 minutes (Figure 1, Table 3). The remaining 68 (53%) videos were not educationally useful, scoring (mean ± SD, 11.1 ± 3.0); the total duration of the videos was 237 minutes. The total viewers of all videos were 2,189,434 while the total viewership/day of videos included in the study was 1,794.5 (average = 29.4; minimum = 1.3; maximum = 932) for useful videos and 1,132.0 (average = 16.6; minimum = 0.6; maximum = 77.9) for videos was not educationally useful. Useful videos were viewed by 1,050,445 viewers (48% of total viewers). Useful videos were created by doctors or professional bodies and they showed a link to an organization such as PACESresources, medicalgallery, and onlinemedicalvideo or the name of the creator and his/her credential. Some videos were linked to universities and known teaching institutes such as the University of Wisconsin School of Medicine and Public Health, the University of Utah, and School of Medicine, Dentistry, and Physician Assistants at Oregon Health & Science University. None of the useful videos were created by pharmaceutical companies (Table 4).

**Table 2:** Videos covering examination of the nervous system on the YouTube

	**Useful videos**	**Videos not educationally useful**
Number of videos (%)	61 (47)	68 (53)
Duration in minutes (%)	253 (52)	237 (48)
Total number of days on YouTube (average, minimum, maximum)	49,824 (816.7869, 78,1643)	59,077 (8687794, 45, 1643)
Total number of viewership (%)	1,050,445 (48)	1,138,989 (52)
Viewership/day (average; minimum; maximum )	1,794.5 (29.4; 1.3; 932)	1,132.0 (16.6; 0.6; 77.9)
Total score (mean ± SD)	14.9 ± 0.2	11.1 ± 3.0

**Table 3:** Characteristics of educationally useful YouTube videos covering nervous system examination

**Item**	**General nervous system**	**Cranial nerves**	**Upper limb**	**Lower limb**	**Balance and coordination**
Number of videos (%)	8 (13)	42 (69)	6 (10)	3 (5)	2 (3)
Duration in minutes (%)	36.4 (14)	155.0 (61)	44.6 (18)	14.0 (6)	3.2 (1)
Total number of days on YouTube (average, minimum, maximum)	3,986 (498.25, 489, 525)	40,421 (962.4048, 78, 1643)	3,513 (585.5, 178, 1280)	872 (290.6667, 178, 516)	1,032 (516, 516, 516)
Total number of viewership (%)	46,010 (4.4)	447,833 (42.6)	541,114 (51.5)	12,541 (1.2)	2,947 (0.3)
Viewership/day (average; minimum; maximum)	91.7 (11.4; 1.3; 45.7)	450.0 (10.7; 1.9; 33.1)	1041.5 (173.5; 3.2; 932)	205.7 (68.5; 12.9; 178)	5.4 (2.7; 2.5; 2.8)
Total score (mean ± SD)	15 ± 0.0	14.95 ± 0.2	15 ± 0.0	15 ± 0.0	15 ± 0.0

**Table 4:** Educationally useful videos on YouTube covering clinical examination of the nervous system

**Title**	**Link**	**Length (min)**	**Date posted**	**Number of viewer**
Abnormal cranial nerves exam: cranial nerve 1-olfaction	http://youtu.be/29wkssonwbA	0.36	March 31,2010	2183
06a.Physical Exam -Upper & Lower Extremities -Part ¼	http://www.youtube.com/watch?v = oezvV-MqSak&feature = channel_video_title	8:31	June 7,2010	1280
06b.Physical Exam -Upper & Lower Extremities -part 2/4	http://www.youtube.com/watch?v = jgwOrpVuTJU&feature = channel_video_title	8:04	June 7, 2010	2804
06c.Physical Exam -Upper & Lower Extremities -part ¾	http://www.youtube.com/watch?v = lPri7qhbB68&feature = channel_video_title	7:56	June 7, 2010	478579
06d.Physical Exam -Upper & Lower Extremities -part 4/4	http://www.youtube.com/watch?v = 9sbQ6K1S2Fs&feature = channel_video_title	9:09	June 7, 2010	43437
01a.Neurologic Exam -part 1.avi	http://www.youtube.com/watch?v = SxgO1Zruu94&feature = channel_video_title	9:41	May 26, 2010	14080
01b.Neurologic Exam -part 2.avi	http://www.youtube.com/watch?v = _YS8m3gyazg&feature = channel_video_title	7:42	May 26, 2010	1245
14.Neurologic Physical Exam -Coordination	http://www.youtube.com/watch?v = mEwZZST-_7E&feature = channel_video_title	2:01	June 4, 2010	1487
15.Neurologic Physical Exam -Gait	http://www.youtube.com/watch?v = 73mHwhq9sP0&feature = channel_video_title	1:10	June 4, 2010	1460
16.Neurologic Exam -Reflexes and Clonus	http://www.youtube.com/watch?v = 5c8UJnNHa44&feature = channel_video_title	5:43	June 4, 2010	7666
17.Neurologic Physical Exam -Sensory & Romberg	http://www.youtube.com/watch?v = ClyXAKkX76I&feature = channel_video_title	10:03	June 4, 2010	3033
18.Neurologic Physical Exam -Motor & Pronator Drift	http://www.youtube.com/watch?v = UmfeLt8-GWY&feature = channel_video_title	8:18	June 4, 2010	3386
18.Neurologic Physical Exam -Motor & Pronator Drift	http://www.youtube.com/watch?v = 0rAfmXg4ZJs&feature = channel_video_title	9:36	June 3, 2010	12751
Medical Gallery- Loyola Full Neurological Exam Part 1	http://www.youtube.com/watch?v = KLY4SEuDsUQ	5:27	July 29, 2010	3722
Medical Gallery- Loyola Full Neurological Exam Part 2.flv	http://www.youtube.com/watch?v = Wk8HQNPl6LM	3:13	July 31, 2010	680
Medical Gallery- Loyola Full Neurological Exam Part 3 .flv	http://www.youtube.com/watch?v = i-r2C1QhyxM	2:15	July 31, 2010	871
Medical Gallery- Loyola Full Neurological Exam Part 4 .flv	http://www.youtube.com/watch?v = 1oSbg1mVUr0	2:48	July 31, 2010	2329
Medical Gallery- Loyola Full Neurological Exam Part 5 .flv	http://www.youtube.com/watch?v = KLYTRNhPChs	4:33	July 31, 2010	22382
Medical Gallery- Loyola Full Neurological Exam Part 6.flv	http://www.youtube.com/watch?v = hJ9vsUAVN_o	0:29	July 31, 2010	719
Medical Gallery- Loyola Full Neurological Exam Part 7.flv	http://www.youtube.com/watch?v = lpjcfzyuDdw	2:35	July 31, 2010	853
Examination of Sensory System of Lower Limb	http://www.youtube.com/watch?v = xLGxwtVa190&feature = channel_video_title	3:14	May 8, 2011	2578
Motor Examination of Lower Limb	http://www.youtube.com/watch?v = BMQpV9jUyT8&feature = channel_video_title	5:06	May 8, 2011	2297
Sensory and Motor Examination of Hand	http://www.youtube.com/watch?v = e6QUCojHKXQ&feature = channel_video_title	2:40	May 8, 2011	585
Examination: Neurology	http://www.youtube.com/watch?v = 7-iNCEP1s4w&feature = related	12:24	May 31, 2011	2779
cranial nerve exam for professionals	http://youtu.be/0wRa9EaBVY4	9:17	February 2, 2011	15535
Cranial Nerve Exam (Part 1 of 3)	http://youtu.be/CEmKFUmuEIk	7:52	January 22, 2010	19278
Cranial Nerve Exam (Part 2 of 3)	http://youtu.be/58kebY7vt8A	10:00	January 25, 2010	10497
Cranial Nerve Exam (Part 3 of 3)	http://youtu.be/Yq-IEw0vfcY	6:56	January 25, 2010	9582
Macleod's Examination of cranial nerve VII	http://youtu.be/cYHFxAtVGgQ	0:46	August 16, 2011	1164
Examination of cranial nerves 9, 10,11, 12	http://youtu.be/jNfcN3riCjY	2:01	August 16, 2011	1201
Neurology Exam .com Cranial Nerves	http://youtu.be/jeB3X4MHOk0	1:44	October 8, 2009	8211
Optic, Occulomotor and abducent Nerve Exam	http://youtu.be/4-yhWLE-ypI	7:03	July 8, 2011	750
V Cranial Nerve Exam	http://youtu.be/XmgbqyfHd_Q	2:36	July 8, 2011	232
VII Cranial Nerve Exam	http://youtu.be/DzPPrAwRth0	2:47	July 8, 2011	529
19.Neurologic Physical Exam -Cranial Nerves	http://youtu.be/0rAfmXg4ZJs	9:36	June 3, 2010	12751
Cranial Nerves	http://youtu.be/pSVw9KsA_mE	3:18	March 9, 2007	13705
Cranial Nerves	http://youtu.be/q1pmFs8qBpY	7:39	November 11, 2009	4789
Neurology - Cranial Nerves III, IV, and VI	http://youtu.be/cuZXz92hd8g	2:15	July 25, 2007	40985
MRCP PACES Seventh Cranial Nerve	http://youtu.be/C48MWa0Eu5k	1:25	February 14, 2009	5840
MRCP PACES Fifth Cranial Nerve	http://youtu.be/nlHEecNiAJ0	2:34	March 5, 2011	1376
MRCP PACES 3,4,,6 Crania Nerves	http://youtu.be/v4OTZeK5E9g	1:18	July 12, 2009	4078
Cranial Nerve VII - sensory (taste) examination	http://youtu.be/bxR-fsfwZS8	0:59	September 6, 2008	3215
Cranial Nerve I - Olfaction 1/25	http://youtu.be/-ksWq5ntmVI	0:52	May 4, 2007	13131
Cranial Nerve II - Visual acuity 2/25	http://youtu.be/1Gs0lJ9Fl2E	1:07	May 4, 2007	11426
Cranial Nerve II- Visual fields 3/25	http://youtu.be/v0LFdvoKPFM	2:31	May 4, 2007	25702
Cranial Nerve II- Fundoscopy 4/25	http://youtu.be/7UhA40RUIpM	0:56	May 4, 2007	16396
Cranial Nerves II & III - Pupillary Light Reflex 5/25	http://youtu.be/iTncbhfbl6A	0:57	May 4, 2007	54425
Cranial Nerves III, IV & VI- Versions 7/25	http://youtu.be/ibD7gH3B-YI	0:34	May 4, 2007	14847
Cranial Nerves III, IV & VI - Ductions 8/25	http://youtu.be/rtMKCtL-FlI	0:54	May 4, 2007	18402
Smooth Pursuit 11/25	http://youtu.be/sKrvQgoR2uk	0:35	May 4, 2007	14030
Cranial Nerve V - Sensory 15/25	http://youtu.be/QGx9_9f84A8	1:02	May 4, 2007	9846
Cranial Nerves V & VII - Corneal reflex 16/25	http://youtu.be/I6ZlzPPAy7c	0:58	May 4, 2007	23568
Cranial Nerve V - Motor 17/25	http://youtu.be/a_t5Jo5mpZI	0:47	May 4, 2007	6338
Abnormal Cranial Nerve V - Motor 17/25	http://youtu.be/yU4h9odugMc	0:51	May 4, 2007	34352
Cranial Nerve VII - Motor 18/25	http://youtu.be/qHHjcVLh2NU	0:52	May 4, 2007	9814
Cranial Nerve VIII - Vestibular 21/25	http://youtu.be/mLA3qi35HP8	0:29	May 4, 2007	5830
Cranial Nerves IX & X - Motor 22/25	http://youtu.be/nDBa1akeHzE	0:42	May 4, 2007	8808
Cranial Nerves 9 & 10- Sensory and Motor: Gag Reflex 23/25	http://youtu.be/_e4LciuRyCA	0:22	May 4, 2007	20769
Cranial Nerve XI - Motor 24/25	http://youtu.be/hPuFMdmkClA	0:52	May 4, 2007	6765
Abnormal Cranial Nerve XII - Motor 25/25	http://youtu.be/AuGkwZwfP2k	1:07	May 4, 2007	34984
Cranial Nerve XII - Motor 25/25	http://youtu.be/qmgdXeRZ20Q	0:52	May 4, 2007	4923
cranial nerve exam	http://youtu.be/pj3efKz4iYg	11:31	April 4, 2011	779
Cranial Nerve Examination	http://youtu.be/jdRJqbB0ZyA	7:35	October 3, 2011	752
MSJC Nursing 234 2010 Cranial Nerve Assessment	http://www.youtube.com/watch?v = oqman6O_-Yw&feature = related	5:48	October 17, 2010	748

Non-educationally useful videos failed due to a number of reasons. The majority of the non-educationally useful videos failed to fulfill one of the major criterion items (45 videos). Among those, over 80% were due to the image lacking clarity or no mention of the creator of the video. Ten videos did not fulfill two major criteria items while nine of the videos fulfilled 3 major criteria items. Three videos did not fulfill 4 criteria items and only one video did not fulfill all the 5 major items.

Not all YouTube videos were accompanied by comments. Some comments have been flagged as spam and others have been removed, as they were inappropriate. A total of 411 comments were found including 124 comments for useful videos and more than double this number, 287 comments, for non-useful videos. This could possibly be due to the negative comments and other critical comments of the examiner and the patient and their interaction. However, most comments were brief, only 1 to 3 words and very few were a full sentence that could be analysed. We enclose examples of these comments, "Glad to hear the aussie accent and humour!!", "Can't tell if they 're both nervous, “him [the examiner] especially, because they're on camera or because a mutual attraction?”, “Too bad we can't find out" and, "Great video! However the plastic hair and the bow tie were a bit disconcerting". However, some of the comments were useful and some raised important points that can help video creators to improve their submission. Examples of these comments are: "I hope to see "real skills" in neurologic assessment and not merely interviewing a patient.”, “I was so disappointed with this one", "He should ask the patient to close her eyes and he has not done that. So be careful of those small mistakes." and, "It looks like he was going to start beating her with the hammer” and “I think the way he elicited a knee reflex was not correct".

## Discussion

The aim of the current study was to conduct an analysis of the YouTube videos covering the nervous system examination. Although several systems that can be used in evaluating videos have been described, [9,19] the system used in this study is simple, easy to apply, covers four key elements, namely, scientific content, technical, authority, and pedagogy parameters. It has also been tested in a previous study assessing videos covering surface anatomy [14]. The findings indicated that during the period from 2 November 2011 to 2 December 2011, videos available on YouTube had approximately 490 minutes of coverage of the nervous system examination of which 253 (52%) can be used for teaching and learning purposes. Videos had a viewership per day on average of 1,794.5 for useful videos and 1,132.0 for non-educationally useful videos. Useful videos were linked to known universities or educational institutes. None of the videos were created by pharmaceutical companies. This indicates the involvement of universities and teaching institutes in promoting the use of educational videos as a resource to learners. This is particularly important with the move of most universities to self-directed learning and student-centered programs. It also reflects the move to the globalization of medicine and knowledge transfer that most universities are currently undertaking as part of their commitment to teaching and learning

There is no doubt that YouTube videos provide time and location flexibility for users. They are available free at no cost and can be of help to students as a learning resource. Furthermore, they allow unlimited access to learning material and hence, learners can practise and review their skills and improvement overtime. These videos do not comprise what takes place in a typical classroom demonstrating clinical examination; YouTube videos are usually brief and self-selected instances focusing on educational principles and key points that need to be highlighted to students.

However, further studies are needed to assess the number of students using YouTube as a resource of knowledge and whether they are able to distinguish between reliable and unreliable electronic resources of knowledge. Non-useful videos failed to fulfill criteria items such as clarity of sounds and images, sound scientific contents, as well as stating the creator of the video. Such critical assessment may be useful to video creators to avoid such problems.

This study also showed that videos covering the examination of balance and coordination and the lower limbs were lacking. The majority of the videos covered examination of cranial nerves, 42 (69%). Such deficiency in certain groups of videos on YouTube necessitates the contribution of universities and colleges of medicine to fill these gaps. In fact, YouTube provides the opportunity for clinical teachers, medical educators and students to share their work with other viewers and be part of the research covering the usefulness of YouTube videos and their uses in education. Although this study represents a snapshot of available resources during 2 November 2011 to 2 December 20110, since then there may be more videos available on nervous system examination. Further studies are needed to assess whether there is an improvement in the quality and coverage of videos on YouTube on this area, and the student’s evaluation of such educational resources [14].

Considering the increasing number of learners using the Internet as their primary source of information [13,20], medical educators and clinical teachers should recognize the importance of YouTube in education and invest in using Web 2.0 in teaching and learning activities such as clinical teaching. This study shows that there are a good number of clinical teachers and educators in addressing the needs of students in clinical examination skills. Educators who are keen to create educational videos should consider planning them and defining the educational objectives of their work [14]. The system used in this paper in evaluating the videos is simple, easy to apply and covers key elements needed in an educational video and can be used by educators interested in creating their videos.

## Conclusions

YouTube provides an adequate resource for learning nervous system examination which can be used by medical students in clinical skills sessions. However, there are a fewer useful videos covering examination of coordination and balance system examination. Useful videos can be used as learning resources to medical students.

## Competing interests

All authors declare that they have not received any support from any organisation for the submitted work. No financial relationships with any organisations that might have an interest in the submitted work and no other relationships or activities that could appear to have influenced the submitted work.

## Authors' contributions

The idea of the project was initiated by SAA. All authors searched the literature and planned the details of the project. SMA, HAA, and RAA tested the criteria individually and the outcomes were checked at different stages by SAA. SAA made a substantial contribution to the writing of the paper and the revision of the final version. All authors read and approved the final version and contributed to it.

## Authors' information

**Samy A. Azer, MD, PhD (USyd), MEd (UNSW), FACG, PHD (UNSW)** is Professor of Medical Education and the Chair of the Curriculum Development and Research Unit, College of Medicine, King Saud University. He was Professor of Medical Education and Chair of Medical Education Research and Development Unit, Faculty of Medicine, Universiti Teknologi MARA, Malaysia. Formerly he was Senior Lecturer in Medical Education at the Faculty of Medicine, Dentistry and Health Sciences, the University of Melbourne and the University of Sydney, Australia. **Sarah M AlEshaiwi, Hala A AlGrain, and Rana A AlKhelaif** are fourth-year medical students at college of medicine, King Saud University, Riyadh, Saudi Arabia.

## Conference presentation

Parts of this work were presented at the Association For Medical Education in Europe (AMEE) Annual Conference incorporating the 4^th^ SIFEM conference which was held in Lyon France, from 25 to 29 August 2012.

## Ethical approval

This project was approved by the Faculty of Medicine Health Research Ethics Committee, King Saud University, Riyadh, Saudi Arabia.

## Pre-publication history

The pre-publication history for this paper can be accessed here:

http://www.biomedcentral.com/1472-6920/12/126/prepub
